# A brief review of membrane vesicles from *Streptococcus mutans*

**DOI:** 10.3389/fmicb.2025.1656926

**Published:** 2025-10-10

**Authors:** Lili Qiu, Qinxia Chen, Gaozhe Zheng, Haiyun Dong, Muxin Xu, Jieyu Zhou, Lingjun Zhang, Yan Sun, Min Wang, Yangyang Pan, Jie Yu, Yihuai Pan, Keke Zhang

**Affiliations:** ^1^Department of Dental Emergency, School and Hospital of Stomatology, Wenzhou Medical University, Wenzhou, China; ^2^School and Hospital of Stomatology, Wenzhou Medical University, Wenzhou, China; ^3^Department of Endodontics, School and Hospital of Stomatology, Wenzhou Medical University, Wenzhou, China; ^4^Department of Pediatric Dentistry, School and Hospital of Stomatology, Wenzhou Medical University, Wenzhou, China

**Keywords:** *Streptococcus mutans*, membrane vesicles, biogenesis, composition, functions

## Abstract

*Streptococcus mutans* (*S. mutans*), a prime conditionally cariogenic organism, produces membrane vesicles (MVs) containing proteins, nucleic acids, and lipids, including cariogenic virulence factors. Factors including culture conditions, peptide signals, bacterial strains, and genes affect the size and contents of MVs. Based on the composition of their contents, MVs play a wide range of roles in self-regulation, microbial interspecies communication, and microbe–host interactions, which have important potential applications in the fields of vaccine research and disease treatment. In this study, we summarize recent developments in the biogenesis, influencing factors, composition, and functions of *S. mutans* MVs to lay a theoretical foundation for their potential clinical application and future research.

## 1 Introduction

*Streptococcus mutans* (*S. mutans*) is an important cariogenic bacterium in the oral cavity that produces various biological factors, such as adhesin and glucosyltransferases (Gtfs), which promote the adhesion and aggregation of other bacteria, ultimately resulting in the formation of a thick biofilm (Banas, 2004). It can produce three types of glucosyltransferases (Gtfs) that utilize sucrose to produce extracellular polysaccharides, which are the main components of the three-dimensional extracellular matrix of plaque biofilms (Ren et al., 2016). In addition, *S. mutans* dynamically releases extracellular deoxyribonucleic acid (eDNA), which strongly interacts with extracellular polysaccharides, synergistically reinforcing microbial adherence and promoting biofilm formation (Klein et al., 2015). In addition, *S. mutans* can effectively assist the biofilm formation and the maintenance of other oral cariogenic microbial species, such as *Candida albicans* (*C. albicans*) and *Lactobacillus* spp., to colonize the tooth surface (Wen et al., 2017; Guo et al., 2021). Taken together, *S. mutans* possesses various mechanisms for forming thick biofilms, which not only benefit its own biofilm formation itself but also promote other microbial biofilms. Combined with its ability to produce and tolerate acid, it eventually promotes a cariogenic environment.

Membrane vesicles (MVs) were first discovered to originate from *Vibrio cholerae* and were considered to be the products of normal physiological processes during bacterial development. Their components were not thoroughly analyzed. This process was initially thought to be related to the excretion of products containing cholera toxins (Chatterjee and Das, 1967). After this initial detection, an increasing number of MVs have been identified in different Gram-negative bacteria, such as *Escherichia coli*, *Burkholderia thailandensis* (*B. thailandensis*), and the periodontal pathogen *Porphyromonas gingivalis* (McBroom et al., 2006; Toyofuku et al., 2019; Sartorio et al., 2021; Wang et al., 2021). Later, researchers investigated whether Gram-positive bacteria could also produce MVs, and several studies found spherical lipid bilayer structures in their supernatants, including *Staphylococcus aureus* (*S. aureus*)*, Bacillus anthracis, Enterococcus faecalis,* and the opportunistic cariogenic bacterium *S. mutans* (Lee et al., 2009; Barnes et al., 2012; Brown et al., 2015). The MVs, all of which were nanostructures with diameters of 20–400 nm, were enclosed by a coated lipid bilayer membrane. The reported components included lipid molecules, nucleotides [such as DNA and ribonucleic acid (RNA)], proteins (such as enzymes and toxins), and immunogenic peptidoglycan (Klimentová and Stulík, 2015; Kroniger et al., 2018; Dell'Annunziata et al., 2021). MVs play vital roles in bacterial growth, proliferation, pathogenicity, bacterial interactions, and microbe–host interactions (Brown et al., 2015; Rainey et al., 2019; Briaud and Carroll, 2020; Juodeikis and Carding, 2022). For instance, MVs derived from *B. thailandensis* display anti-biofilm effects on *S. mutans*, whereas MVs from *S. aureus* have been shown to enhance the development of airway hypersensitivity to inhaled allergens (Bitto et al., 2020; Wang et al., 2021).

The first successful extraction of MVs from a supernatant culture solution of *S. mutans* was reported in 2014 (Liao et al., 2014). In this study, classical vesicular structures of MVs were identified in cell-free supernatants using uranyl acetate staining (Liao et al., 2014). This study also revealed that MVs from *S. mutans* actively released DNA to assist in autologous biofilm formation (Liao et al., 2014). Subsequently, they have been reported in an increasing number of studies. Recent studies have demonstrated that *S. mutans* MVs harbor nucleic acids, proteins, and lipids, including multiple cariogenic virulence factors that may be involved in self-regulation, microbial interspecies communication, and microbe–host interactions (Iwabuchi et al., 2021; Rainey et al., 2019). This review focuses on the biogenesis, composition, and functions of *S. mutans* MVs. We aim to provide a theoretical basis for future research on *S. mutans* MVs through this mini-review.

## 2 *Streptococcus mutans* MV biogenesis

All Gram-positive bacteria have a 20–40 nm thick cell wall, which aids in resisting0 extreme conditions such as strong osmotic pressure changes, DNA-damaging agents, antibiotics, and some toxic chemical reagents (Liu et al., 2009; Bose et al., 2020). Peptidoglycan, a major component of the cell wall, in addition to polysaccharides and proteins, acts as a barrier that blocks the release of MVs. Current research indicates that MV biogenesis within Gram-positive bacteria occurs through either autolysin-dependent or endolysin-dependent pathways (Toyofuku et al., 2017; Abe et al., 2021).

**Figure 1:**
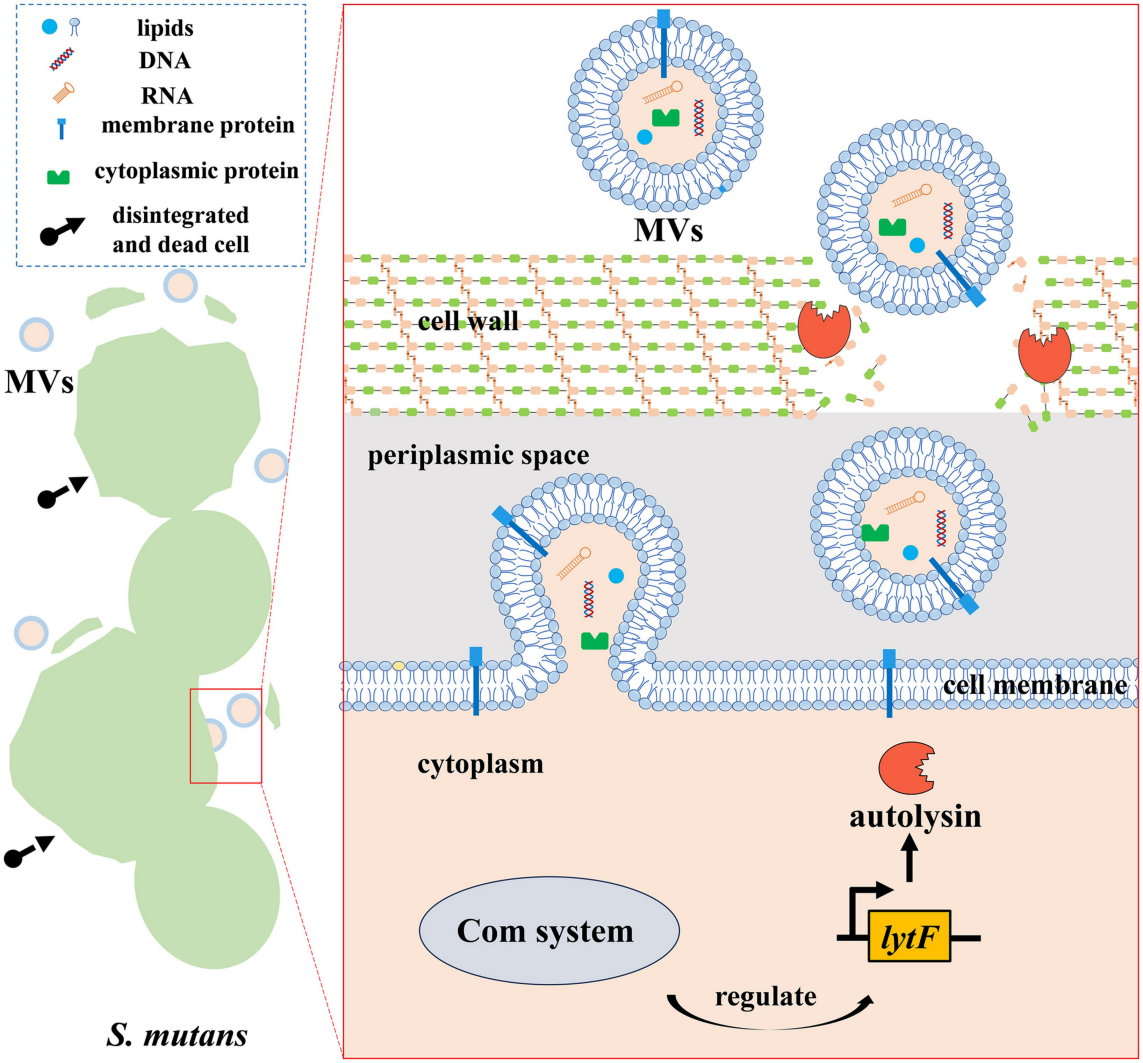
*S. mutans* MV biogenesis. MV biogenesis in *S. mutans* appears to occur in an autolysin-dependent manner. The *lytF*-encoding autolysin can be regulated by the Com system of *S. mutans*. Autolysin, a peptidoglycan hydrolase, facilitates the release of MVs by increasing the porosity of the cell wall, finally triggering cells’ disintegration and death.

In the autolysin-dependent process, the extent of peptidoglycan cross-linking and autolysin activity regulate MV production by altering the permeability of the cell wall in Gram-positive bacteria (Abe et al., 2021). As peptidoglycan hydrolases, autolysins facilitate the release of MVs by increasing the porosity of the thick Gram-positive cell wall. They always localize to the septum, where they exhibit peptidoglycan hydrolase activity, leading to the isolation of MVs from bacteria (Abe et al., 2021). For example, *S. aureus* can promote the fluidity of its cytoplasmic membrane using modulins, followed by the breakdown of peptidoglycan through autolysins, which can be encoded by *sle1* and *atl*, leading to the release of MVs (Wang and Lee, 2024). Recent research has indicated that *S. mutans* can release MVs through an autolysin-dependent mechanism, although the details of this process remain largely unclear (Figure 1). Specifically, *S. mutans* MVs are released via a cell-to-cell communication system mediated by peptide signals called the Com system (Nagasawa et al., 2025). It can regulate the expression of the autolysin (LytF)-encoding gene *lytF* to further control the release of autolysins, which further modulate the production of MVs (Nagasawa et al., 2025). Electron microscopy images indicated that MV release was accompanied by cell death in a subpopulation of cells, which benefited the remaining cells (Nagasawa et al., 2025). Moreover, it is worth noting that the autolysin AtlA (encoded by *altA*) with peptidoglycan-degrading activity is likely a major contributor to the biogenesis of *S. mutans* MVs and is readily detectable within these vesicles (Morales-Aparicio et al., 2020). However, further studies are required to identify their specific roles during the process of *S. mutans* MV biogenesis.

Recent studies have revealed that, in addition to autolysins, phage-derived endolysins can induce MVs in Gram-positive bacteria. The expression of endolysin, which is encoded by a defective prophage, triggers vesicle formation and release in Gram-positive bacteria (Toyofuku et al., 2017). Similar to the explosive cell lysis observed in Gram-negative bacteria, the enzymatic action of endolysins weakens peptidoglycan, causing bacterial contents protrude outward and be released as MVs in certain Gram-positive bacteria. Another group of bacteria undergoes a process called “bubbling cell death,” which results from a loss of cell integrity, and this also leads to the release of MVs (Toyofuku et al., 2023). More research is required to further explore whether *S. mutans* can produce MVs through this mechanism, although no associated genes have been found in *S. mutans* to date (Nagasawa et al., 2025).

## 3 Factors affecting *Streptococcus mutans* MVs

MV biogenesis is a highly regulated and active process (Brown et al., 2014; Lee et al., 2018). Several factors have been implicated in affecting *S. mutans* MVs, including culture conditions, peptide signals, bacterial strains, and gene regulation.

### 3.1 Culture conditions

pH and culture medium have been reported as two culture conditions that affect *S. mutans* MVs. First, the properties of *S. mutans* MVs are regulated by pH (Cao et al., 2020; Wen et al., 2021; Iwabuchi et al., 2021). In particular, the initial pH of the culture environment appears to play an important role in MV biogenesis. MVs prepared from *S. mutans* under different initial pH conditions exhibited different sizes. Although particles of 0–200 nm^2^ dominated in MVs extracted from both pH 6.0 and pH 8.0 culture media, a higher proportion of MVs exceeding 1,000 nm^2^ was found under alkaline conditions at pH 8.0 (Iwabuchi et al., 2021). Another study reported similar trends, where the diameter of *S. mutans* MVs at pH 7.5 was significantly larger than that at pH 5.5 (Cao et al., 2020). In addition, biofilm formation triggered by the treatment with different MVs from *S. mutans* under various initial pH conditions exhibited different results; these different MVs affected the structure and characteristics of the *S. mutans* biofilm (Iwabuchi et al., 2021). Although the specific mechanism may require further investigation, it is clear that MVs under different pH conditions are significantly different, not only in size but also in content. *S. mutans* produces larger MVs under neutral conditions (pH 7.5), despite harboring approximately 10-fold less protein content (standardized by bacterial colony-forming units) compared to acidic conditions (pH 5.5) (Cao et al., 2020).

In addition to pH, the culture medium also influences the characteristics of *S. mutans* MVs (Nagasawa et al., 2025). Brain heart infusion (BHI) is a complex medium that is commonly used for oral bacterial cultures, including *S. mutans*. Interestingly, the response of *S. mutans* to autologous SigX (alternative sigma factor)-inducing peptide (XIP) is restricted by this type of medium. In contrast, chemically defined medium (CDM) is a peptide-free culture medium that supports *S. mutans* growth and limits the function of self-generated competence-stimulating peptides (CSPs) (Son et al., 2012). Proteins within *S. mutans* MVs from the BHI medium and CDM were found to be quite different (Nagasawa et al., 2025). Moreover, *S. mutans* wild-type MVs isolated from BHI could induce the biofilm formation of *S. mutans* Δ*gtfBC*, a strain that lacks the corresponding coding products, glucosyltransferase B (GtfB) and glucosyltransferase C (GtfC), and could barely form biofilms (Nagasawa et al., 2025). In contrast, *S. mutans* wild-type MVs isolated from CDM have limited effects on the biofilm formation of *S. mutans* Δ*gtfBC* (Nagasawa et al., 2025).

### 3.2 Peptide signals

In the autolysin-dependent process of *S. mutans* MV biogenesis, MV release occurs via a cell-to-cell communication system mediated by peptide signals, called the Com system (Nagasawa et al., 2025). The Com system consists of an upstream CSP-mediated ComDE pathway and a downstream XIP-involved ComRS pathway, which can regulate sigX to further control the autolysin-encoding gene *lytF*, the product of which is responsible for *S. mutans* MV release by targeting peptidoglycans (Khan et al., 2016; Nagasawa et al., 2025). Therefore, CSPs with 18 amino acids or XIP with seven amino acids may contribute to *S. mutans* MV biogenesis. As expected, the exogenous addition of CSP or XIP to BHI or CDM promoted *S. mutans* MV formation compared to the corresponding medium without peptide signals (Nagasawa et al., 2025). In addition, both CSP and XIP altered the protein contents of *S. mutans* MVs isolated from the corresponding medium (Nagasawa et al., 2025).

### 3.3 Bacterial strains

Different strains of *S. mutans* can produce different numbers of MVs (Wen et al., 2021). For instance, according to a quantitative analysis, *S. mutans* 27–3, a clinical strain isolated from a patient with active caries, can produce approximately 8-fold more MVs than *S. mutans* UA159 under the same conditions (Wen et al., 2021). Moreover, the above results are in line with qualitative observations from transmission electron microscopy (TEM), which revealed many more vesicular structures surrounding the cells of *S. mutans* 27–3 than *S. mutans* UA159 (Wen et al., 2021). The whole genome sequencing of *S. mutans* 27–3 revealed significant differences compared to *S. mutans* UA159, including the addition of 192 genes and the deletion of 275 genes. This may be related to the increase in MV yields (Wen et al., 2021). Evidence suggests that these genes are implicated in *S. mutans* MV biogenesis.

### 3.4 Genes

In addition to culture conditions, peptide signals, and bacterial strains, the properties of *S. mutans* MVs, including size, quantity, and content, are regulated by specific genes.

The *lytF-*encoding product, LytF, has been reported to induce cell death in a subpopulation of *S. mutans* and to promote eDNA production (Nagasawa et al., 2020). Interestingly, *lytF*-expressing *S. mutans* cells were abundant near the base of the biofilm, while all cells within the biofilm received the CSP signal, which could induce the expression of *lytF* (Nagasawa et al., 2020). *S. mutans* MV biogenesis has been reported to occur in an autolysin-dependent manner; the autolysin (LytF)-encoding gene *lytF* can undoubtedly affect *S. mutans* MVs. The *S. mutans* Δ*lytF* strain produced fewer MVs than its wild-type strain under CSP or XIP treatment. Moreover, the defect of the *S. mutans* Δ*lytF* strain in producing MVs was restored in a *lytF*-complemented strain. These results confirm the involvement of *lytF* in *S. mutans* MV biogenesis (Nagasawa et al., 2025).

GtfB and GtfC are encoded by *gtfB and gtfC*, respectively, and they are two of the most important glycosyltransferases involved in insoluble glucan synthesis in *S. mutans*. These two important cariogenic virulence factors are present in *S. mutans* MVs, as revealed by anti-GTF antiserum (Senpuku et al., 2019). These two GTF-encoding genes influence *S. mutans* MV biogenesis through multiple pathways. First, MVs isolated from strains deficient in *gtfB* and *gtfC* display markedly different effects on autologous and other oral microbial biofilm formation compared to those from the wild-type strain. The effects of *S. mutans* MVs were restricted significantly under *S. mutans* Δ*gtfBC* MV. For example, the remarkable enhancement effects of MVs from the wild-type strain on *S. mutans* UA 159 biofilm formation were not found in MVs from the *S. mutans* Δ*gtfBC* strain (Senpuku et al., 2019). The significant repression effects of MVs from wild-type *S. mutans* on the biofilm formation of *Streptococcus gordonii* (*S. gordonii*) and *Streptococcus sanguinis* (*S. sanguinis*) were lost when the MVs were replaced with the ones from the *S. mutans* Δ*gtfBC* strain (Cui et al., 2022). The protein content of MVs was decreased in *S. mutans* Δ*gtfC* and Δ*gtfBC* strains compared to MVs from the wild-type strain. In contrast, MVs from *S. mutans* Δ*gtfB* had a similar protein concentration compared to the wild-type strain. In addition, *S. mutans* and its Δ*gtfB* strain had larger MVs than Δ*gtfC* and Δ*gtfBC* strains (Nakamura et al., 2020). These results prove that GtfC, but not GtfB, influences the protein content and size of *S. mutans* MVs (Nakamura et al., 2020).

SMU*_*833, a putative glycosyltransferase encoded by *smu_833*, is recognized as an important virulence factor in *S. mutans*. The deficiency of *smu_833* resulted in no changes in the overall biofilm biomass, but it caused changes in biofilm architecture, decreased acidogenesis *in vitro*, and reduced virulence in a rat caries model (Rainey et al., 2019). In addition, it can alter the interactions between eDNA and glucan, the two primary biofilm matrix constituents (Jakubovics and Burgess, 2015). The deficiency of *smu_833* led to a reduction in glucan levels, which resulted from a decrease in Gtfs (GtfB and GtfC) and enhanced eDNA generation. Notably, the increase in eDNA was accompanied by improved release of MVs. The increase in eDNA and MVs as a result of the *smu_833* deletion appears to compensate for the defects in Gtfs, making up for any biofilm biomass changes to some extent (Rainey et al., 2019).

Furthermore, SrtA, encoded by *srtA*, is a transpeptidase that covalently combines several surface-associated proteins with peptidoglycans within the cell wall and has been reported to play a role in MV biogenesis in *S. mutans* (Liao et al., 2014). The lack of *srtA* in *S. mutans* impairs the membrane localization and activity of the multifunctional adhesin P1 and other proteins, which subsequently affects bacterial adhesion and weakens biofilm formation. Therefore, SrtA is a significant protein that plays a role in biofilm formation (Liao et al., 2014). However, subsequent experiments showed that *srtA* deficiency did not disrupt the production of MVs significantly, as supported by transmission electron microscope observations (Liao et al., 2014). Quantitative analysis from another study suggested that the *srtA* deficiency strain had a higher MV particle concentration than the wild type (Morales-Aparicio et al., 2020). The protein profile of MVs was significantly altered by *srtA* deficiency (Liao et al., 2014; Morales-Aparicio et al., 2020). Detailed analysis using Western blotting revealed that MVs extracted from the *S. mutans ΔsrtA* strain produced lower levels of adhesin P1, glucan-binding proteins B (GbpB) and C (GbpC), and Gtfs compared to MVs released by the wild-type strain (Liao et al., 2014). In addition to differences in content, physical properties of Δ*srtA* MVs, analyzed by nanoparticle tracking analysis, displayed a larger mean diameter than the wild-type MVs (Morales-Aparicio et al., 2020). Overall, *srtA* in *S. mutans* not only affects MV quantity but also the protein component and size (Liao et al., 2014; Morales-Aparicio et al., 2020).

Similar to SrtA, the 4′-phosphopantetheinyl transferase Sfp has been reported to affect MV biogenesis. Sfp deficiency by *sfp* mutation in other Gram-positive bacteria impairs the production of MVs and results in defects in biofilm formation (Brown et al., 2014). In *S. mutans*, the *sfp* homolog *mubP* (*smu_1334c*) is located within a prevalent large genomic island called TnSmu2 and affects MV biogenesis (Wu et al., 2010). In contrast to *srtA*, *sfp* deficiency results in lower MV particle concentration compared to its wild type (Morales-Aparicio et al., 2020). In addition, proteomic analyses have shown that *sfp* mutation also affects the protein composition of MVs (Morales-Aparicio et al., 2020; Wen et al., 2021). This indicates that protein transport from bacteria to MVs is selective and active, and multiple factors may affect this process during different delivery phases (Wen et al., 2021). In addition, the diameter of Δ*sfp* MVs differed from that of wild-type strain MVs (Morales-Aparicio et al., 2020; Wen et al., 2021).

The OpuB transporter, encoded by *opuB,* was shown to play a critical role in the biogenesis of MVs and affected the composition of *S. mutans* MVs. For biogenesis, the *opuB*-deficient (Δ *opuB*) strain produced smaller and more MVs than *S. mutans* UA159 at pH 7.5 (Wang C. et al., 2025; Wang W. et al., 2025). However, there was no significant difference in MV quantity or size when the *opuB*-deficient strain was compared to the wild type at acidic pH 5.5 (Wang C. et al., 2025; Wang W. et al., 2025). When *S. mutans* MV composition was examined, the knockout of *opuB* impacted the lipid concentration and composition of MVs (Wang C. et al., 2025; Wang W. et al., 2025). In addition, 108 and 279 proteins in MVs were altered by more than 2-fold in the *opuB*-deficient strain under pH 7.5 and pH 5.5 conditions, respectively (Wang C. et al., 2025; Wang W. et al., 2025). Genes currently reported to affect *S. mutans* MVs are listed in Table 1.

**Table 1:** Representative genes affecting *S. mutans* MVs.

Genes	Protein	Regulation	Reference
*lytF*	LytF	Quantity and content	Nagasawa et al. (2025)
*gtfB*	GtfB	Content	Nakamura et al. (2020) and Senpuku et al. (2019)
*gtfC*	GtfC	Content and size	Nakamura et al. (2020)
*smu_833*	SMU_833	Quantity and content	Rainey et al. (2019)
*srtA*	SrtA	Quantity, content, and size	Liao et al. (2014) and Morales-Aparicio et al. (2020)
*sfp*	Sfp	Quantity, content, and size	Morales-Aparicio et al. (2020) and Wen et al. (2021)
*opub*	OpuB	Content and size	Wang C. et al. (2025) and Wang W. et al. (2025)

In addition to the factors discussed above, other elements involved in *S. mutans* MV biogenesis need to be investigated. It is clear that components of *S. mutans* MV-related genes and their regulation play a role in *S. mutans* MV biosynthesis. Therefore, composition analysis and identification of *S. mutans* MVs may contribute to the control of MV biogenesis.

## 4 Composition of *Streptococcus mutans* MVs

Recently, increasing research attention has been devoted to the content of *S. mutans* MVs, mainly focusing on proteins, lipids, and nucleic acids.

### 4.1 Proteins

*S. mutans* MVs contain many proteins, and the MV protein content has been adopted as a measurement standard to quantify MVs (Cao et al., 2020). Proteomic analyses have identified proteins within MVs that are associated with several biological processes (Cao et al., 2020). A total of 509 proteins were detected in *S. mutans* MVs, comprising 351 proteins at pH 5.5 and 495 proteins at pH 7.5 (Cao et al., 2020). Although MVs with smaller sizes had significantly higher protein content (normalized by bacterial colony-forming units) under acidic conditions (pH 5.5) compared to pH 7.5, 344 proteins were detected at both pH 5.5 and pH 7.5. They included metabolic enzymes, membrane transporters, secretory proteins, signal peptidase, proteases, structural components of the ribosome, cell wall-associated hydrolases, and lysozymes (Cao et al., 2020). Notably, many virulence factors of *S. mutans*, such as Gtfs, surface protein antigen P1 (SpaP), glucan-binding proteins (Gbps), lactate dehydrogenase (LDH), and dextranase (DexA), have been identified in MVs using proteomic analysis (Cao et al., 2020).

In addition to the regulatory effects on the protein content due to pH changes, the deletion of some genes, such as *srtA* and *spf,* undoubtedly changes the protein composition and quantity of MVs, as mentioned above (Cao et al., 2020; Morales-Aparicio et al., 2020). It was reported that MVs from the *S. mutans* Δ*sfp* strain shared 61.16% protein similarity with its wild-type strain MVs (Morales-Aparicio et al., 2020). The similarity in MV protein composition was 28.10% when the *S. mutans* Δ*srtA* strain was compared to its wild-type strain (Morales-Aparicio et al., 2020). Comparatively, these two strains with gene mutations shared 28.51% similarity in MV protein composition (Morales-Aparicio et al., 2020). These results illustrate that the transport of proteins to *S. mutans* MVs is a selective process that is substantially influenced by the presence of SrtA, and to a lesser extent, by Sfp (Cao et al., 2020; Morales-Aparicio et al., 2020). All proteins reported under different conditions are listed in Table 2.

**Table 2:** Selected upregulated proteins in *S. mutans* MVs under different conditions.

Condition	Upregulated proteins	Reference
*S. mutans* MV proteins at pH 5.5 compared to pH 7.5	GbpD, GbpA, GtfD, GtfB, TpiA, Pgk, LeuS, Gap, SMU_689, KxYKxGKxW signal peptide-containing protein, RelA, PfkA, GapC, putative hydrolase SMU_367, IlvC, GapA_2, and LysS	Cao et al. (2020)
Proteins in *S. mutans* MVs compared to the corresponding cytoplasmic membrane	SMU_1904c*, PotD*, SMU_367*, FruA*, GbpD*, Ftf, GbpC, GbpB, AtlA, SMU82_1213c, GtfC, SpaP, GtfB, SMU_63c, DexA, GtfD, SMU_1733c, and SMU_609	Morales-Aparicio et al. (2020)
Proteins in *S. mutans* Δ*srtA* MVs compared to the corresponding cytoplasmic membrane	SMU_1904c*, GbpC, Ftf, AtlA, GbpB, GtfC, DexA, SMU82_1213c, and GtfB	Morales-Aparicio et al. (2020)
Proteins in *S. mutans* Δ*sfp* MVs compared to the corresponding cytoplasmic membrane	SMU_963c*, SMU_1904c*, SMU_172*, SMU_367*, BacA2*, BacA*, WapE*, FruA*, GbpD, GbpB, AtlA, GbpC, Ftf, GtfD, SMU_63c, GtfC, SMU82_1213c, GtfB, and BrpA	Morales-Aparicio et al. (2020)

*MVs only.

### 4.2 Lipids

Lipids are vital structural constituents of bacterial cell membranes, including *S. mutans*, through which MVs are secreted (Morales-Aparicio et al., 2020). Hence, it is clear that outer membrane phospholipids are components of *S. mutans* MVs. Nevertheless, some lipids are found exclusively in MVs and not in the outer membrane of *S. mutans* (Morales-Aparicio et al., 2020). Lipids in MVs from *S. mutans* were analyzed using liquid chromatography-mass spectrometry, and approximately 30 individual lipids were identified in *S. mutans* MVs (Morales-Aparicio et al., 2020). The relative proportion of each lipid category varies between the cytomembrane and MVs. The analysis showed that cardiolipins and flavonoids are present in higher proportions in MVs compared to the cytomembrane (Morales-Aparicio et al., 2020). The lipid architecture plays an important role in environmental adaptation (Fozo and Quivey, 2004). The richness of monounsaturated long-chain fatty acids helps *S. mutans* improve its tolerance to the acidic environment generated during the fermentation of carbohydrates into organic acid end products (Morales-Aparicio et al., 2020). Interestingly, in MVs from *S. mutans* Δ*srtA* and Δ*sfp* strains, the level of monounsaturated long-chain fatty acids was significantly increased; this transformation may assist these strains to tolerate the acidic environment (Morales-Aparicio et al., 2020).

### 4.3 Nucleic acids

MVs from Gram-positive bacteria have been reported to harbor nucleic acids such as DNA and RNA, which can be delivered to other bacteria and facilitate horizontal gene transfer (HGT) (Schooling et al., 2009; Díaz-Garrido et al., 2021).

To identify whether *S. mutans* MVs can act as carriers for the release of eDNA, hydrolyzed MVs were used to detect eDNA existence. Unsurprisingly, the experiments confirmed the presence of eDNA in MVs (Liao et al., 2014). eDNA plays a crucial role in biofilm formation, including that of its own and several other bacteria (Senpuku et al., 2019; Wu et al., 2020). Several studies have also identified RNA in MVs, which are important for some physiological processes (Munhoz da Rocha et al., 2020). These RNAs include messenger RNA (mRNA), ribosomal RNA (rRNA), transfer RNA (tRNA), and long non-coding RNA (lncRNA) (Munhoz da Rocha et al., 2020). A recent study identified tRNA in *S. mutans* MVs that could facilitate cell proliferation together with the migration of the oral mucosa, support focal adhesion complex formation within organoids, and aid wound healing in a mouse model (Oh et al., 2025). RNA sequencing analysis identified “microRNA-like” molecules in *S. mutans*, suggesting that these RNAs may contribute to bacteria that are analogous to microRNAs within eukaryotes (Lee and Hong, 2012; Munhoz da Rocha et al., 2020).

In addition to the studies on MV components discussed above, further research on MV composition and its influencing factors is needed to further understand MVs and explore their potential functions and applications.

## 5 Functions of *Streptococcus mutans* MVs

**Figure 2:**
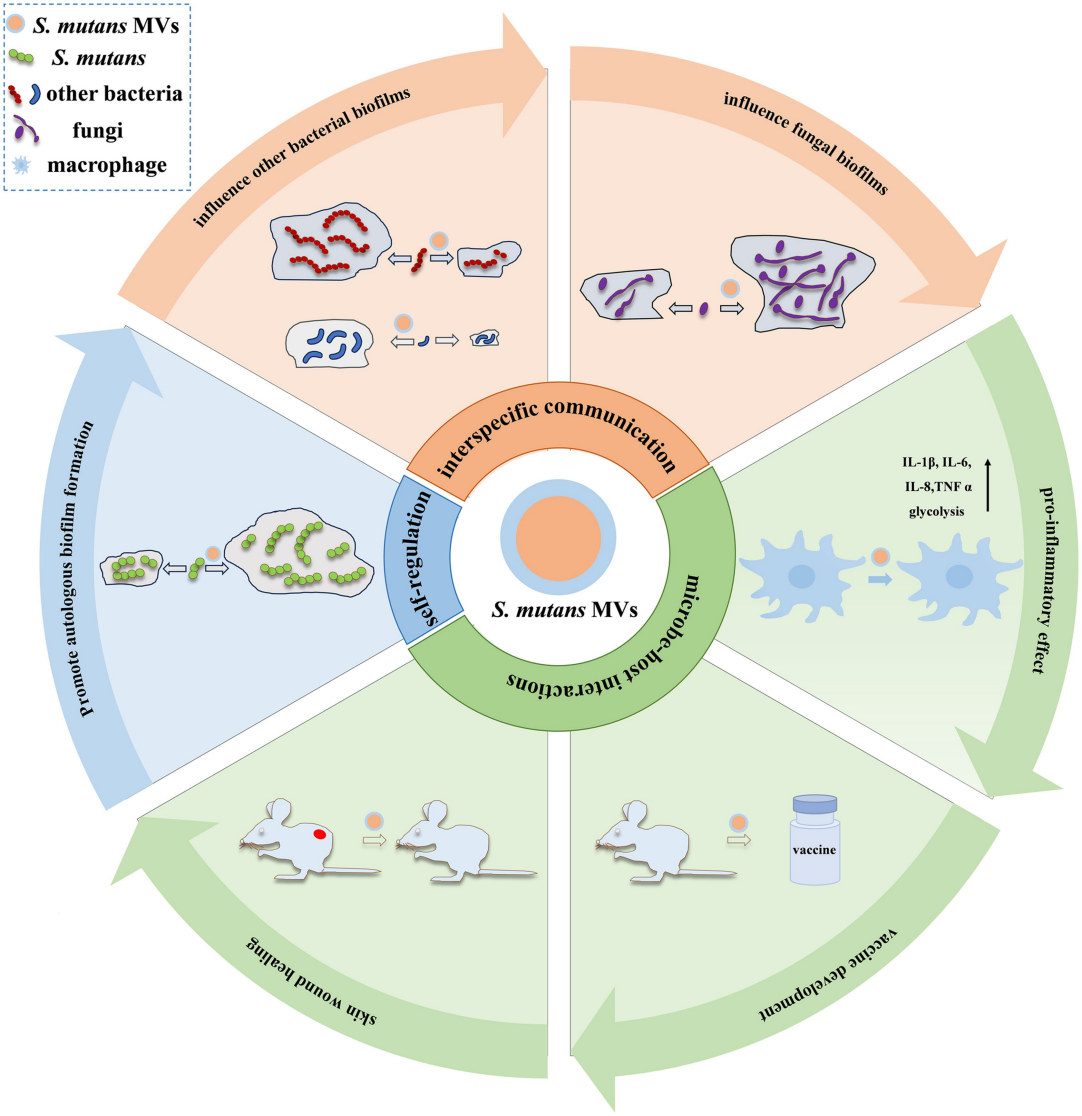
Functions of *S. mutans* MVs. Currently, major research advances in *S. mutans* MV functions have focused on self-regulation, microbial communication, and microbe–host interactions. The self-regulatory effect of *S. mutans* MVs has been mainly observed in autologous biofilm formation (Nakamura et al., 2020). In interspecies communication, *S. mutans* MVs display suppression (such as *S. gordonii* and *S. sanguinis*) (Cui et al., 2022) or enhancement (such as *S. sanguinis*, *S. mitis*, *S. oralis*, *A. naeslundii*, *S. gordonii,* and *A. oris*) (Senpuku et al., 2019) effects on bacterial biofilms and augment fungal biofilm development, such as *C. albicans* (Wu et al., 2020). In microbe–host interactions, *S. mutans* MVs have been reported to elevate the release of inflammatory cytokines (such as IL-1β, IL-6, IL-8, and TNF-*α*) in macrophages and induce cellular glycolysis (Song et al., 2024); stimulate an immune response to produce anti-Gtfs antibodies, which make the development of vaccines feasible (Nakamura et al., 2020); and promote skin wound healing in mice (Oh et al., 2025).

Owing to the different compositions of MVs, it is possible that they perform different functions. However, many of these potential functions, based on their contents, have not yet been verified. Currently, major research advances have focused on self-regulation, microbial communication, and microbe–host interactions (Figure 2).

### 5.1 Self-regulation

The self-regulatory effect of *S. mutans* MVs has been mainly observed in autologous biofilm formation. A previous study showed that MVs derived from *S. mutans* contribute to autologous biofilm formation (Senpuku et al., 2019). The extracellular biofilm matrix of *S. mutans* mainly consists of glucan polysaccharides, eDNA, and lipoteichoic acid (Klein et al., 2015). A significant part of this process is the glucan matrix, which facilitates *S. mutans* adherence to the tooth surface, maintains mechanical stability, protects microorganisms from environmental assaults, reserves energy sources, limits the diffusion of substances into and out of the biofilm, and helps concentrate metal ions and other physiological nutrients (Schilling and Bowen, 1992; Koo et al., 2009). The glucan matrix of *S. mutans* is synthesized and organized using extracellular Gtfs. Surprisingly, one of the most important components in MVs secreted from *S. mutans* is Gtfs, which is a key enzyme in dental caries development (Nakamura et al., 2020). Several studies have suggested that *S. mutans* secretes MVs harboring Gtfs that can augment sucrose metabolism and promote autologous biofilm formation. Compared to MVs from the *S. mutans* Δ*gtfBC* strain, the MVs from the wild-type strain significantly enhanced biofilm formation (Senpuku et al., 2019). Interestingly, GtfB largely adheres to the MV surface (Nakamura et al., 2020). In contrast, GtfC is primarily present within MVs and regulates MV size and aggregation and *S. mutans* biofilm formation (Nakamura et al., 2020). Another factor that plays an important role in the formation of *S. mutans* biofilms on tissues within the oral cavity is eDNA, which contributes to adhesion and the accumulation of *S. mutans,* as well as the architecture and stability of autologous biofilms (Castillo Pedraza et al., 2017; Kim et al., 2018). To investigate the function of eDNA and MVs in *S. mutans* biofilm formation, Senpuku et al. reported the extraction of a purified complex consisting of DNA and MVs with Gtfs from *S. mutans* and incubation with the *S. mutans* Δ*gtfBC* strain. Interestingly, the results suggested that this complex induced Gtfs-dependent *S. mutans* Δ*gtfBC* biofilm formation (Senpuku et al., 2019). Moreover, short DNA fragments associated with *S. mutans* MVs can significantly promote autologous biofilm formation (Senpuku et al., 2019). MVs that had eDNA removed showed a different effect on *S. mutans* Δ*gtfBC* biofilm formation compared to MVs at a relatively low concentration (Senpuku et al., 2019). Therefore, it is inferred that MVs can assist in autologous biofilm formation, further enhancing the cariogenicity of *S. mutans* and promoting dental caries.

### 5.2 Interspecies communication

MVs are natural carriers of molecules that are protected by them, allowing long-distance delivery of these biological molecules and avoiding direct intercellular contact to safely reach their final destination (Gill et al., 2019). This characteristic endows *S. mutans* MVs with the function of interspecies communication by influencing biofilm formation in other species.

A recent study showed that MVs not only contribute to the biofilm formation of *S. mutans* but also influence the formation of other bacterial biofilms. *S. sanguinis* and *S. gordonii* are the initial colonizers of tooth surfaces. They compete with *S. mutans* for hydrogen peroxide (H_2_O_2_) and are countered by *S. mutans* through mutacin (Zhang et al., 2025). It has been reported that biofilm formation by *S. gordonii* and *S. sanguinis* is inhibited by *S. mutans* MVs, where the Gtfs in MVs play a role (Cui et al., 2022). When co-cultured with MVs from *S. mutans*, *S. gordonii,* and *S. sanguinis,* biofilm formation was significantly suppressed (Cui et al., 2022). In contrast, *S. mutans* Δ*gtfBC* MVs had no significant effect on the biofilm formation of these two species (Cui et al., 2022). In addition, *S. mutans* MVs suppressed the expression of their virulence genes, including *GtfG* (encoding glucosyltransferase in *S. gordonii*)*, GtfP* (encoding glucosyltransferase in *S. sanguinis*), and *SpxB* (encoding pyruvate oxidase to produce H_2_O_2_) (Cui et al., 2022). Another study reported that biofilm formation by *S. sanguinis*, *Streptococcus mitis*, *Streptococcus oralis*, *Actinomyces naeslundii*, *S. gordonii,* and *Actinomyces oris* can be facilitated by *S. mutans* MVs (Senpuku et al., 2019). Further studies have shown that this facilitative action is GtfB- and GtfC-dependent, except in *A. naeslundii*, where MVs from *S. mutans* Δ*gtfBC* still display promotional effects (Senpuku et al., 2019). The differing effects of *S. mutans* MVs on *S. gordonii* and *S. sanguinis* biofilms observed in the two separate studies may have resulted from differences in culture conditions, bacterial strains, and experimental methods.

In addition to being involved in the communication between bacteria, *S. mutans* MVs also affect fungi, such as *C. albicans*, one of the most common colonizers within the oral cavity. MVs derived from *S. mutans* can augment the biofilm development of *C. albicans* and are Gtf-dependent (Wu et al., 2020). In addition, *S. mutans* MVs enhance the pathogenicity and carbohydrate metabolism of *C. albicans*. The enhanced pathogenicity of fungal biofilms was revealed in a bovine dentin demineralization experiment, where *S. mutans* MV-containing groups showed greater hardness loss, more exposure, and increased damage to dentin tubules (Wu et al., 2022). Promoted carbohydrate metabolism is mainly revealed by the increase in related metabolites and protein expression (Wu et al., 2022). In addition, when co-cultured with MVs, *C. albicans* biofilms have a three-dimensional structure with an abundant extracellular matrix, and *C. albicans* forms hyphal cells under biofilm-forming conditions (Wang C. et al., 2025; Wang W. et al., 2025).

Other potential functions of *S. mutans* MVs involved in interspecies communication include providing substrates for horizontal gene transfer (HGT) and regulating gene expression and protein translation, which are based on nucleic acid loading in MVs. HGT has recently been identified as an effective mechanism for microbiomes to interact, helping bacteria acquire new genetic traits in addition to plasmids (Arnold et al., 2022). The distribution of antimicrobial resistance genes is an example of this interaction and is considered a type of HGT (Yaron et al., 2000). *S. mutans* has been reported to release eDNA via MVs into developing biofilms; therefore, it is reasonable to infer that MVs from *S. mutans* also offer other competent bacteria an important source of transformation through this novel mechanism (Roberts and Kreth, 2014; Campoccia et al., 2021). In addition to eDNA, RNA is another type of nucleic acid found in MVs. As mentioned previously, multiple types of RNA can be delivered by *S. mutans* MVs (Munhoz da Rocha et al., 2020). It may contribute to bacterial communication by regulating gene expression via non-coding RNAs and protein translation via messenger RNAs (Munhoz da Rocha et al., 2020). The effects of RNA within *S. mutans* MVs have been demonstrated in microbe–host interactions (Tsatsaronis et al., 2018; Oh et al., 2025). However, whether nucleic acids within *S. mutans* MVs are involved in HGT, microbial interspecies regulation of gene expression, and protein translation requires further investigation.

### 5.3 Microbe–host interactions

The role of *S. mutans* MVs in microbe–host interactions is mainly reflected in their immunity. On the one hand, *S. mutans* MVs can trigger an immune response and induce a pro-inflammatory effect. On the other hand, *S. mutans* MVs have emerged as promising tools for the development of vaccines and immunotherapeutic strategies against infectious and non-infectious diseases (Nakao et al., 2011; Long et al., 2022).

A recent study showed that *S. mutans* MVs could notably elevate the release of inflammatory cytokines and induce macrophage glycolysis. When cultured with *S. mutans* MVs, the expression of macrophage pro-inflammatory cytokines, such as interleukin-1β (IL-1β), interleukin-6, interleukin-8, and tumor necrosis factor *α*, was significantly increased (Song et al., 2024). Among these highly expressed cytokines, IL-1β was particularly prominent, and its increased production induced by *S. mutans* MVs could occur through the activation of the nucleotide-binding oligomerization domain-like receptor protein 3 (NLRP3), absent in melanoma 2 (AIM2), apoptosis-associated speck-like protein containing CARD (ASC), and nucleotide-binding oligomerization domain-like receptor C4 (NLRC4) inflammasomes (Song et al., 2024). In addition, potassium ion efflux and adenosine triphosphate generation were involved in IL-1β production induced by *S. mutans* MVs (Song et al., 2024). Macrophage glycolysis is a crucial part of this pro-inflammatory process for classical activation (Song et al., 2024). In addition to these two main findings, *S. mutans* MVs promoted *S. mutans* colonization of oral epithelial cells and suppressed macrophage phagocytosis against *S. mutans* (Song et al., 2024).

In addition to their pro-inflammatory effects, *S. mutans* MVs can induce an immune response in the oral environment, making vaccine development feasible (Nakao et al., 2011; Long et al., 2022). GtfB and GtfC, which are closely associated with *S. mutans* MVs, are key cariogenic virulence factors that contribute to biofilm formation by themselves and through other microorganisms (Liao et al., 2014; Senpuku et al., 2019). Based on this evidence, *S. mutans* MVs are regarded as crucial virulence factors and targets for biofilm-associated disease control. It was reported that MVs from *S. mutans* wild-type, Δ*gtfB*, Δ*gtfC*, and Δ*gtfBC* strains produced anti-MV IgA and IgG antibodies after intranasal immunization of mice (Nakamura et al., 2020). Further investigation revealed that it is the antibodies induced by MVs from *S. mutans* wild-type and *S. mutans* Δ*gtfB* strains, rather than *S. mutans* Δ*gtfC* and *S. mutans* Δ*gtfBC* strains, that react with MV Gtfs (Nakamura et al., 2020). It is clear that *S. mutans* MVs harboring GtfC are operative mucosal immunogens that induce anti-Gtf antibody production (Nakamura et al., 2020). Using *S. mutans* MVs as antigens stimulated IgA and IgG antibody generation against to Gtfs successfully, which may be useful for future vaccine development. However, considering the extremely complex composition of *S. mutans* MVs, the potential side effects of this process require further investigation.

In addition to the two proven effects discussed above, *S. mutans* MVs have been shown to play a role in skin wound healing in mice (Oh et al., 2025). Specifically, *S. mutans* MVs not only promoted the proliferation of human oral organoids, assisted in the migration of oral epithelial cells, and enhanced the formation of focal adhesion complexes but also facilitated wound healing in the dorsal skin of mice (Oh et al., 2025). Further research has revealed that tRNA variants, the most abundant RNAs within *S. mutans* MVs, play a vital role in this process (Oh et al., 2025). Surprisingly, the tRNA mentioned above could take effect even when electroporated into *Escherichia coli* MVs, in addition to being packaged within *S. mutans* MVs (Oh et al., 2025). Further research revealed that the promotion of skin wound healing occurred through a Toll-like receptor 3-dependent mechanism (Oh et al., 2025). This study demonstrated that the use of *S. mutans* MVs and RNA cargo is a promising therapeutic strategy for skin wound rehabilitation (Oh et al., 2025).

## 6 Conclusion and future perspectives

In summary, *S. mutans* MVs containing multiple molecules, including virulence factors, have been recognized as powerful tools for *S. mutans* to survive and compete. As demonstrated above, they have diverse capabilities, including self-regulation, microbial interspecies communication, and microbe—host interactions. Further systematic and comprehensive clarification of MV biogenesis, composition, and function, not limited to *S. mutans*, will help us better understand the potential of MVs. Although the characteristics of MVs make the management of biofilm-associated diseases even more challenging, they provide a potential target for the control of these diseases. In addition, the MV-based development of vaccines or therapeutics is an important direction for future research. MVs can provide a protected environment for carrying this cargo, thereby demonstrating great potential as a tool for drug delivery.
